# Transcriptome Analysis of Peritoneal Cells Reveals the Early Immune Response of Flounder (*Paralichthys olivaceus*) to Inactivated *Vibrio anguillarum* Immunization

**DOI:** 10.3390/vaccines11101603

**Published:** 2023-10-16

**Authors:** Xianghu Meng, Heng Chi, Zuobing Zhang, Qian Li, Xiuzhen Sheng, Xiaoqian Tang, Jing Xing, Wenbin Zhan

**Affiliations:** 1 Laboratory of Pathology and Immunology of Aquatic Animals, KLMME, Ocean University of China, Qingdao 266003, China; mxh6008@stu.ouc.edu.cn (X.M.); lq19980901@163.com (Q.L.); xzsheng@ouc.edu.cn (X.S.); tangxq@ouc.edu.cn (X.T.); xingjing@ouc.edu.cn (J.X.); wbzhan@ouc.edu.cn (W.Z.); 2 Function Laboratory for Marine Fisheries Science and Food Production Processes, Qingdao National Laboratory for Marine Science and Technology, Qingdao 266071, China; 3 College of Life Sciences, Shanxi University, Taiyuan 030006, China; zbzhang@sxu.edu.cn

**Keywords:** peritoneal cells, RNA-seq, immune response, fish

## Abstract

*Vibrio anguillarum* (*V. anguillarum*) is a bacterium that seriously harms flounder and other aquaculture species. Vaccination is an effective means of preventing vibriosis and is mainly administered by intraperitoneal injection. Effective antigen processing at the initial stage of immunization is essential to elicit adaptive immune responses and improve vaccine efficacy. To understand the early immune response of flounder caused by inactivated *V. anguillarum*, we detected the transcriptome profiles of the cells in the peritoneal cavity (*Po*PerCs) after inactivated *V. anguillarum* immunization. More than 10 billion high-quality reads were obtained, of which about 89.33% were successfully mapped to the reference genome of flounder. A total of 1985, 3072, 4001, and 5476 differentially expressed genes were captured at 6, 12, 24, and 48 h post immunization, respectively. The hub module correlated with the immunization time was identified by WGCNA. GO and KEGG analysis showed that hub module genes were abundantly expressed in various immune-related aspects, including the response to stimuli, the immune system process, signal transducer activity, autophagy, the NOD-like receptor signaling pathway, the toll-like receptor signaling pathway, the T cell receptor signaling pathway, and Th17 cell differentiation. Additionally, genes related to Th cell differentiation are presented as heatmaps. These genes constitute a complex immune regulatory network, mainly involved in pathogen recognition, antigen processing and presentation, and Th cell differentiation. The results of this study provide the first transcriptome profile of *Po*PerCs associated with inactivated *V. anguillarum* immunity and lay a solid foundation for further studies on effective *V. anguillarum* vaccines.

## 1. Introduction

The flounder (*Paralichthys olivaceus*), an economically important marine fish, is mainly cultured in the north of China [1]. Vibriosis is a deadly hemorrhagic septicaemic disease that continuously damages flounder culture, causing severe negative consequences for industrial production [2]. *Vibrio anguillarum* (*V. anguillarum*), a Gram-negative, rod-shaped bacterium, is one of the main causative agents of vibriosis in many marine fish species, including various species of economic importance in the aquaculture industry, such as sea bass (*Dicenthrarchus labrax*), carp (*Cyprinus carpio*), rainbow trout (*Oncorhynchus mykiss*), Atlantic salmon (*Salmo salar*), Atlantic cod (*Gadus morhua* L.), ayu (*Plecoglossus altivelis*), turbot (*Scophthalmus maximus*), and flounder (*Paralichthys olivaceus*) [3,4,5,6].

To prevent vibriosis caused by *V. anguillarum*, many studies have been carried out, which have largely focused on developing safe and efficient *V. anguillarum* vaccines [7,8,9]. Vaccines, as safe and environmentally friendly biological agents, offer numerous advantages compared to other biological approaches, including precise targeting and durable immune protection [10]. Currently, although vaccines have evolved from traditional inactivated and live vaccines to subunit vaccines and nucleic acid vaccines (DNA vaccines and RNA-based vaccines), inactivated vaccines still represent the predominant type of licensed Vibrio vaccines [11]. We all know that inactivated vaccines are produced from intact infectious bacteria or viruses, which are rendered non-pathogenic through physical or chemical methods after amplification and cultivation, while still preserving their antigenicity. Inactivated vaccines are safe for use, easily stored, and can enable recipients to generate humoral immune responses, while posing no risk of contamination [12]. The administration of inactivated vaccines primarily occurs through intraperitoneal injection (i.p.), and this process can be accurately simulated using computer mathematical models, enabling improved optimization of vaccination dosage and timing [11,13]. Teleosts possess resident peritoneal cavity cells (PerCs) including lymphocytes, granulocytes, macrophages, and dendritic cells, which play a role in antigen presentation and pathogen clearance in the early stages of immunity or infection [14]. Elucidating the mechanism of inactivated *V. anguillarum* after i.p. delivery, especially the role of PerCs, for the cells first exposed to foreign antigens, would contribute to the optimization of vaccination, and therefore deserves intensive studies.

Transcriptome analysis is a powerful tool for uncovering the relationship between gene responses and external stimuli, leading to a better understanding of the underlying molecular mechanisms of host–pathogen interactions [15]. It has been employed to investigate immune-related genes and signaling pathways in the head kidney and spleen of teleosts after immunization with inactivated vaccines. For instance, bivalent-inactivated *Aeromonas salmonicida* and *V. anguillarum* vaccines have been shown to activate the rainbow trout complement system and up-regulate *TCRα*, *T-bet*, and *HSP90* in the head kidney [16]. Additionally, differential genes in the Atlantic salmon spleen elicited by immunization with inactivated *Aeromonas salmonicida* vaccine were significantly enriched in cytokine–cytokine receptor interaction, the MAPK signaling pathway, PI3K-Akt signaling pathway, and TNF signaling pathway [17]. However, transcriptome analysis of PerCs from teleosts after immunization has not been reported.

In this study, our objective was to identify key genes in flounder PerCs (*Po*PerCs) associated with the initial stages of inactivated *V. anguillarum* immunization at the transcriptional level. We identified hub modules using WGCNA and analyzed their biological functions and signaling pathways through GO and KEGG. Furthermore, we presented the key genes of the main KEGG pathway in the form of heat maps. This study enhances our understanding of the immune response of *Po*PerCs induced by inactivated *V. anguillarum* and provides a valuable reference for optimizing inactivated *V. anguillarum* vaccines.

## 2. Materials and Methods

### 2.1. Ethics Statement

The use of fish in this study strictly adhered to the recommendations outlined in the Guidelines for the Use of Experimental Animals of Ocean University of China. The protocol for animal care and handling used in this study was approved by the Committee on the Ethics of Animal Experiments of Ocean University of China (permission number: 20190101). Before injection and sampling procedures, the fish were anesthetized by using 100 mg/L MS222 (Sigma, St. Louis, MO, USA). Every effort was made to minimize any potential suffering and ensure the well-being of the animal.

### 2.2. Experimental Fish 

Healthy flounders, 25–30 cm in length, were obtained from a farm in Qingdao, Shandong Province, China. These fish were acclimated in tanks filled with aerated seawater at 20 °C and were fed commercial feed. Prior to the experiment, flounders were randomly selected and underwent standard microscopic and bacteriological examinations to ensure they were not infected with *V. anguillarum*. 

### 2.3. Inactivated Bacteria Immunization and Sample Collection

*V. anguillarum* strain had been previously isolated from diseased flounder and was stored in our laboratory [18]. Inactivated *V. anguillarum* was prepared as previously described [13]. Briefly, *V. anguillarum* resuspended in 0.01 M phosphate-buffered saline (PBS) with a pH of 7.2 to reach a concentration of 1 × 10^9^ CFU/mL was treated with 0.5% formalin at 30 °C for 48 h. After being washed three times with PBS, the inactivated bacteria suspension was adjusted to a concentration of 1.0 × 10^8^ CFU/mL and stored at 4 °C until use.

For immunization and RNA-seq analysis, each fish was injected intraperitoneally with 100 μL of inactivated bacteria suspension. The *Po*PerCs were sampled from nine fish at each of the specified time points (0, 6, 12, 24, 48 h) after immunization, with three fish combined as a separate sample. These sampling groups were labeled IV-0 (control), IV-6, IV-12, IV-24, and IV-48. All collected cells were immediately frozen in liquid nitrogen and subsequently stored at −80 °C.

In accordance with the specified time points and sampling strategies, the intestine, spleen, head kidney, and peripheral blood leukocytes (PBLs) were isolated to detect the expression of Th cell differentiation-related genes in various body compartments, including the blood, mucosae, and lymphoid organs. The isolation procedure for *Po*PerCs and PBLs was carried out as previously described [19,20]. All samples were promptly frozen in liquid nitrogen and stored at −80 °C until RNA isolation.

### 2.4. RNA Extraction, Library Construction, and Sequencing

Total RNA was extracted from each sample using the TRIzol Reagent Kit (Invitrogen, Carlsbad, CA, USA) following the manufacturer’s instructions. The quality and integrity of the RNA were assessed using a nanodrop spectrophotometer (Nanodrop, Wilmington, NC, USA) and an Agilent 2100 Bioanalyzer (Agilent, Santa Clara, CA, USA). Total mRNA was enriched using Oligo(dT) beads. Subsequently, the enriched mRNA was fragmented into short fragments using a fragmentation buffer and reverse transcribed into cDNA with the NEBNext Ultra RNA Library Prep Kit for Illumina (NEB, Ipswich, MA, USA). Purified double-stranded cDNA fragments underwent end repair, had an A base added, and were ligated to Illumina sequencing adapters. The ligation reaction was purified with the AMPure XP Beads (1.0×). Ligated fragments were size selected through agarose gel electrophoresis and polymerase chain reaction (PCR) amplification. The resulting cDNA library was sequenced using an Illumina Novaseq6000 by Gene Denovo Biotechnology Co. (Guangzhou, China).

### 2.5. Transcript Assembly and Correlation Analysis of Samples

The reads obtained from the sequencer consist of raw data that may include adaptors or low-quality bases, thereby impacting subsequent assembly and analysis. To address this, we employed fastp (version 0.18.0) [21] to filter out low-quality reads containing adapters or having more than 10% unknown nucleotides (N), as well as reads consisting of 50% or more low-quality bases (Q-value ≤ 20). Subsequently, we conducted Q20, Q30, and GC-content calculations using the resulting clean data. The clean reads were then aligned to the flounder reference genome using HISAT2.2.4 [22] with the default parameters and the addition of “-rna-strandness RF”. 

We performed correlation analysis using the R package to evaluate the reproducibility between samples. Correlation coefficients were computed to assess the level of reproducibility between the two parallel experiments, with a correlation coefficient closer to 1 indicating higher reproducibility.

### 2.6. Differentially Expressed Genes and WGCNA Analysis

FPKM (fragments per kilobase of transcript per million mapped reads) values were calculated for each transcribed region using RSEM software to quantify the gene expression abundance and variation [23]. Differentially expressed gene (DEG) analysis between two distinct groups was carried out using DESeq2 software (version 1.40.2) [24], and genes with *p* values below 0.05 and an absolute fold change ≥ 2 were classified as differentially expressed genes.

The weighted gene co-expression network was constructed using the WGCNA v1.47 package in R [25], where genes exhibiting similar expression patterns were grouped into modules. Co-expression modules were constructed using the blockwiseModules function with default settings, except for the power set to 8, TOMType set as unsigned, and minModuleSize set to 50. Correlation analysis was conducted using the module eigengene in relation to post immunization times. Modules exhibiting a Pearson correlation coefficient (*R*) > 0.5 and a *p*-value < 0.05 were deemed to be strongly correlated with the inactivated *V. anguillarum* immunization times. These modules showing a high correlation with immunization time were designated as hub modules.

### 2.7. GO and KEGG Enrichment Analysis

The hub module genes underwent GO and KEGG enrichment analysis. GO enrichment analysis within the hub modules identified significantly enriched genes associated with specific biological functions [26]. KEGG analysis within the hub module identified metabolic pathways or signal transduction pathways significantly enriched among the hub module genes [27]. The identification of significantly enriched GO terms and KEGG pathways was conducted through a hypergeometric test with a threshold of *p* ≤ 0.05.

### 2.8. Quantitative Real-Time PCR (qPCR) 

To validate the reliability of the transcriptome data, we selected T helper (Th) cell differentiation-related genes (*NOD2*, *TLR2*, *MHC-II*, *ATG5*, *GABARAPL1*, *TCRβ*, *CD4-1*, *STAT4*, and *RORα*) for qPCR analysis using the Light-Cycler^®^ 480 II Real-Time System (Roche, Basel, Switzerland). Each reaction comprised 10 μL of 2 × Universal SYBR Green Fast qPCR Mix, 2 μL of cDNA template, 0.4 μL each of forward and reverse primers, and 7.2 μL of DEPC water. The reaction protocol consists of a pre-denaturation at 95 °C for 3 min, followed by 40 cycles of annealing at 95 °C for 5 s, and an extension at 60 °C for 30 s. All reactions were carried out in triplicate. The expression levels of these genes were analyzed using the 2^−ΔΔCt^ method with *β-actin* serving as the internal control. The information about primers is provided in Table 1.

### 2.9. Statistical Analysis

Statistical analysis was performed by using one-way ANOVA and Tukey’s multiple comparison tests in IBM Statistical Product and Service Solutions (SPSS) Statistics for Windows, version 20.0 (IBM, Armonk, NY, USA). The data are presented as mean ± SD, and statistical significance was defined as *p* < 0.05.

## 3. Results

### 3.1. Transcriptome Sequencing Quality

**Figure 1:**
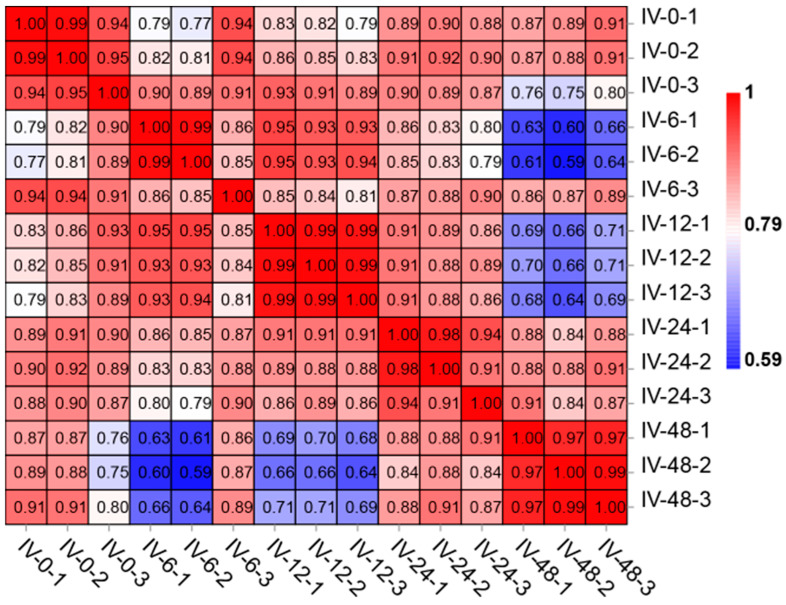
Pearson correlation coefficients for comparisons among all samples. The closer the number is to 1, the darker the red color, indicating the stronger the sample correlation.

A total of 120,880,346,400 raw data were generated by the transcriptome sequencing of 15 *Po*PerCs samples (see Table 2). After removing low-quality data, each sample retained between 6,600,187,229 and 10,459,182,090 clean data reads; these clean reads exhibited Q20 and Q30 values higher than 97.16% and 92.38%, respectively. The average GC content across all samples was 47.74%. The unique mapping rate for each sample within the flounder reference transcriptome ranged from 89.33% to 90.33%, with a total mapping rate exceeding 91.15%. Moreover, the Pearson correlation coefficients (*R*) for the relationships between samples within each group exceeded 0.85 (see Figure 1), indicating a high level of reproducibility among samples within each group. These results confirm the acquisition of high-quality transcriptome data, suitable for further analyses.

### 3.2. Differentially Expressed Genes at Different Times after Immunization

**Figure 2:**
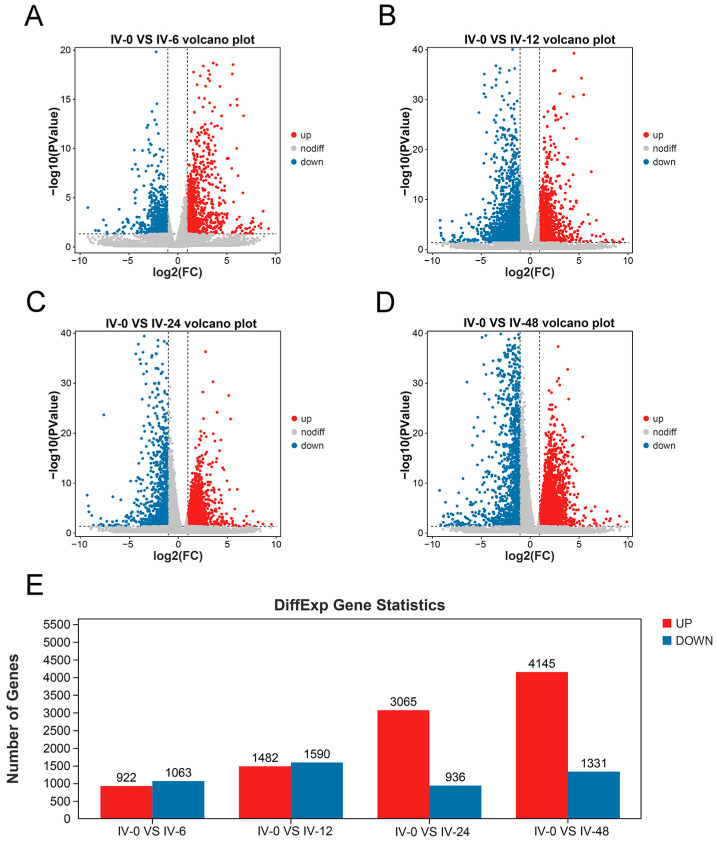
Differentially expressed genes between the control group and the four treatment groups. (**A**) IV-0 vs. IV-6. (**B**) IV-0 vs. IV-12. (**C**) IV-0 vs. IV-24. (**D**) IV-0 vs. IV-48. (**E**) Statistical plot of DEGs for four times points. The DEGs were screened based on |log2(fold change)| ≥ 2 and *p* < 0.05.

As shown in Figure 2A–D, volcano plots were used to visualize the differentially expressed genes (DEGs) between the control group (IV-0) and the four treatment groups (IV-6, IV-12, IV-24, and IV-48). The total DEGs identified in the IV-0 vs. IV-6, IV-0 vs. IV-12, IV-0 vs. IV-24, and IV-0 vs. IV-48 comparisons were 1985 (922 up-regulated and 1063 down-regulated), 3072 (1482 up-regulated and 1590 down-regulated), 4001 (3065 up-regulated and 936 down-regulated), and 5476 (4145 up-regulated and 1331 down-regulated), respectively (Figure 2E).

### 3.3. Gene Co-Expression Modules Post Immunization

**Figure 3:**
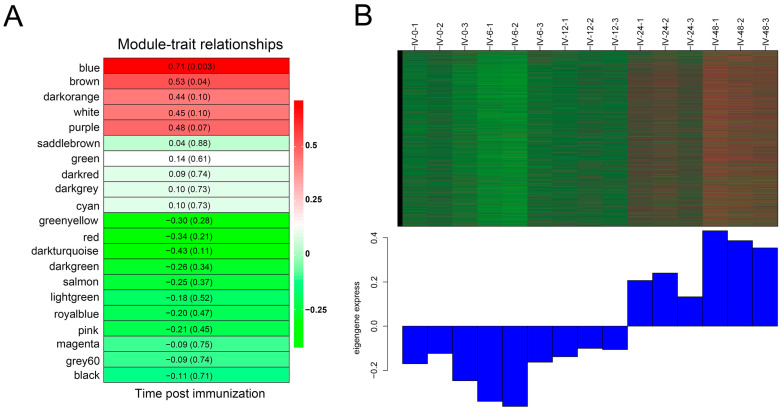
The features of hub modules. (**A**) Module–immunization time weight correlations and corresponding *p*-values and *R*-values. (**B**) Heat map and the eigengene expression of blue module.

A total of 21 co-expression modules, each containing 95 to 11,616 genes, were identified through WGCNA based on the analysis of 20,814 genes from 15 samples (see Table 3). The association between module eigengene and immunization time was assessed to determine their correlations. The blue (*R* = 0.71, *p* = 0.003) modules demonstrated a significant correlation with immunization time (Figure 3A), and were therefore selected as hub modules for further analysis. Specifically, the expression of most genes within the blue module eigengene was up-regulated at 24 h and 48 h post-immunization (Figure 3B).

### 3.4. Enrich Classification Hub Modules Genes

**Figure 4:**
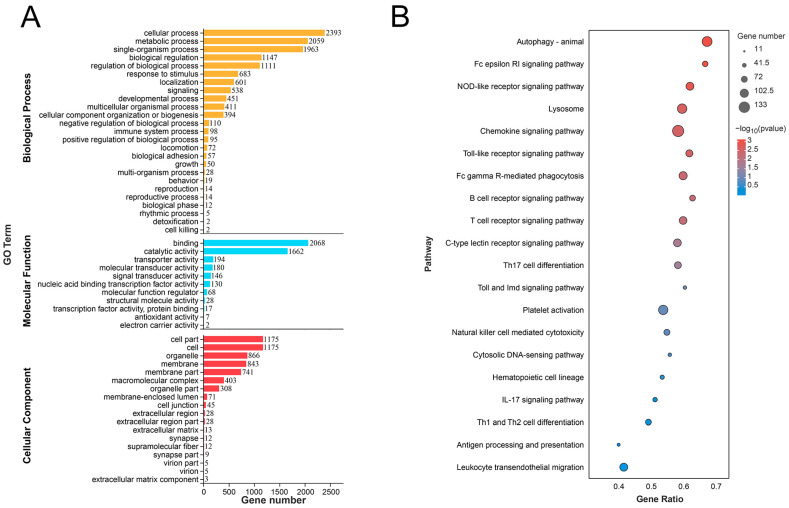
Enrichment analysis of hub module. (**A**) GO enrichment analysis of hub module (*p* < 0.05). (**B**) KEGG enrichment bubble plot of immune-related pathways in hub module (*p* < 0.05).

To uncover the functions of hub module genes and the immune-related pathways they are involved in, GO and KEGG enrichment analyses were performed for these hub module genes. The Go terms were mainly classified into three groups: biological process (BP), molecular function (MF), and cellular component (CC). As shown in Figure 4A, the BP category contained hub genes associated with the cellular process, response to stimulus, signaling, and immune system process, along with other processes. The MF category included hub genes involved in binding, catalytic activity, signal transducer activity, and nucleic acid binding transcription factor activity. In the CC category, hub genes were related to cellular components such as cells, membranes, extracellular regions, and macromolecular complexes. The KEGG enrichment results revealed the top 20 immune-related pathways for blue module (Figure 4B). Genes within the blue module were significantly enriched in pathways such as autophagy, the NOD-like receptor signaling pathway, the toll-like receptor signaling pathway, the T cell receptor signaling pathway, and Th17 cell differentiation (*p* < 0.05).

### 3.5. Expression of T Helper (Th) Cell Differentiation-Related Genes

**Figure 5:**
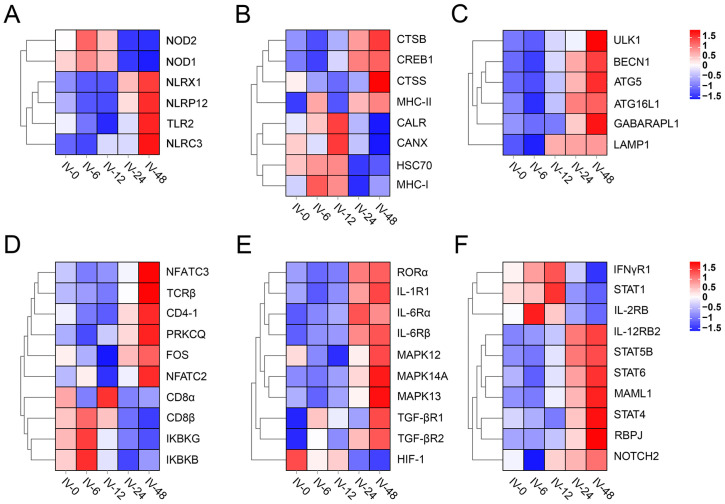
Heat map of Th cell differentiation-related pathway genes in blue modules; color shade represents the expression level of genes. (**A**) Pattern recognition receptors. (**B**) Antigen processing and presentation. (**C**) Autophagy. (**D**) T cell receptor signaling pathway. (**E**) Th 17 cell differentiation. (**F**) Th1 and Th2 cell differentiation pathway.

Based on KEGG annotations, we further screened genes related to the pattern recognition receptor (PRR), autophagy, antigen processing and presentation (APP), the T cell receptor signaling pathway, Th17 cell differentiation, as well as Th1 and Th2 cell differentiation. In the PRR category, genes such as *NLPX1*, *NLPR12*, *NLRC3*, and *TLR2* exhibited up-regulated expression at 48 h post-immunization, whereas *NOD1* and *NOD2* showed up-regulated expression at 6 h (Figure 5A). Hub genes in the MHC-I pathway included *HSC70*, *MHC-I*, *CALR*, and *CANX*. The expression of these genes as well as their downstream genes (*CD8α*, *CD8β*, and *IFNγR1*) exhibited earlier up-regulated genes involved in the MHC-II pathway and Th cell differentiation (see Figure 5B,D–F). These latter genes included *MHC-II*, *STAT4*, *STAT5B*, *STAT6*, *CD4-1*, *TCRβ*, *NFATC*, and *RORα*. Interestingly, the expression of genes related to autophagy such as *BECN1*, *ULK1*, *ATG5*, *ATG16L1*, and *GABARAPL1* also exhibited up-regulation at either 24 or 48 h post-immunization (see Figure 5C).

### 3.6. Validation of Gene Expression Profiles by qPCR

**Figure 6:**
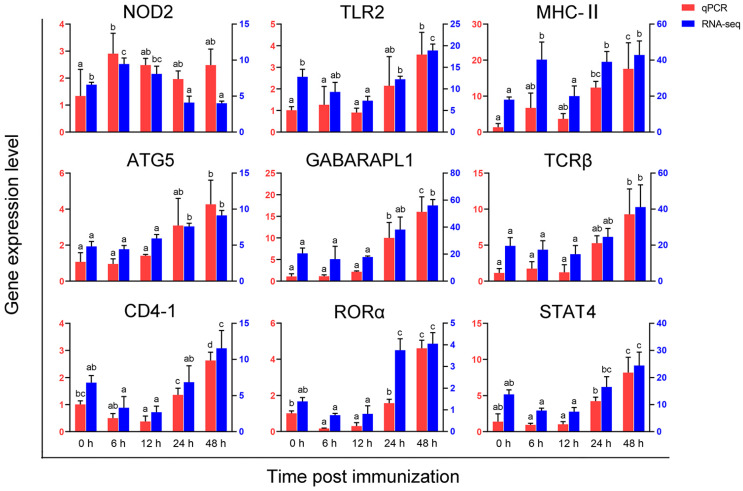
Validation of Th cell differentiation-related genes by qPCR. The expression patterns of 9 genes at 0, 6, 12, 24, and 48 h after immunization were determined by qPCR. The red columns represent the qPCR values, while the blue columns represent the fpkm values of RNA-seq. Results are shown as means ± SD (N = 3). Different letters on the bar represent significant differences (*p* < 0.05) of difference between the experimental (6, 12, 24, and 48 h after immunization) and control group (0 h after immunization).

The expression profiles of *NOD2*, *TLR2*, *MHC-II*, *ATG5*, *GABARAPL1*, *TCR β*, *CD4-1*, *STAT4*, and *RORα* was further examined by qPCR, as these genes are closely related to the PRR, antigen processing and presentation, and Th cell differentiation. The results largely confirmed expression patterns of the selected genes in RNA seq (see Figure 6). Specifically, the expression of *TLR2*, *ATG5*, *GABARAPL1*, *TCRβ*, *CD4-1*, *STAT4*, and *RORα* was significantly up-regulated at 48 h after immunization (*p* < 0.05). The expression of NOD2 was significantly up-regulated at 6 h post-immunization, followed by down-regulation at both 24 and 48 h (*p* < 0.05). Moreover, *MHC-II* exhibited significant up-regulation at 6, 24, and 48 h after immunization (*p* < 0.05). The expression profiles of these genes in intestine, spleen, head kidney and peripheral blood leukocytes (PBLs) is illustrated in Figure S1. These findings indicated that genes related to Th cell differentiation were expressed to various degrees in the blood, mucosae, and lymphoid organs within 48 h following the inactivation of *Vibrio anguillarum*.

## 4. Discussion

In the early stages of vertebrate immunity, antigen processing occurs through both innate and adaptive immunity [28]. Serving as key participants in both innate and adaptive immune responses, PerCs play an important role in the early stage of i.p. immunization [13]. In this study, we conducted a transcriptome analysis to investigate the immune response of *Po*PerCs from flounder following the immunization with inactivated *V. anguillarum* with a focus on the first 48 h. The RNA-Seq data generated a total of 119,496,253,411 high-quality clean reads, and these reads were well annotated, benefiting from the flounder’s whole genome sequence [29]. These data provide a valuable resource for exploring the genetic landscape of *Po*PerCs and an in-depth understanding of gene expression profiles post-immunization with inactivated *V. anguillarum.* Furthermore, these findings lay a strong foundation for further vaccine-related research. The reliability and accuracy of RNA-seq data were confirmed by qPCR results, which can be used for subsequent analyses. We identified 14,561 DEGs, with 7210 DEGs (comprising 75% of all up-regulated DEGs) exhibiting up-regulation at 24 h and 48 h post-immunization. To further screen out the hub genes following immunization with inactivated *V. anguillarum*, we conducted WGCNA resulting in a total of 21 co-expression modules. One of these modules exhibited a significant correlation with the immunization time, and its gene expression pattern closely resembled that of DEGs, with up-regulation at 24 h and 48 h post-immunization. Consequently, the genes within this module were designated as hub genes for subsequent analysis. The identified immune-related hub genes were primarily associated with pathways such as NOD-like receptor signaling, toll-like receptor signaling, autophagy, lysosome, antigen processing and presentation, T cell receptor signaling, and Th17 cell differentiation.

The initial step in mounting an effective immune response is the timely recognition of the pathogen. Typically, the innate immune system identifies pathogen-associated molecular patterns (PAMPs) through a series of pattern recognition receptors (PRRs) [30]. Our study identified a total of six classical PRR genes associated with the immunization of *V. anguillarum*, namely, NOD1, NOD2, NLRX1, NLRP12, NLRC3, and TLR2. Previous studies have reported that NOD1 is capable of responding to Gram-negative bacteria and may play a crucial role in recognizing LPS [31]. Further studies have demonstrated that muramyl dipeptide (MDP), a peptidoglycan motif found in both Gram-negative and Gram-positive bacteria, can effectively activate NOD2 [32]. In mammals, NLRX1, NLRP12, and NLRC3 have been identified as negative regulators of the NF-κB signaling pathway [33]. In our study, after immunization with inactivated *V. anguillarum*, the expression of *NLRX1*, *NLRP12*, and *NLRC3* was down-regulated at 6 h and 12 h, while the expression of *IKBKB* and *IKBKG* genes showed the opposite trend (see Figure 5D). IKBKB and IKBKG belong to the nuclear factor kB (IKB) kinase (IKK) family, which can lead to ubiquitination and protease resolution of IKB, ultimately resulting in the release and activation of NF-κB [34]. Therefore, NLRX1, NLRP12, and NLRC3 are likely to perform similar regulatory functions from fish to mammals. TLR2 in teleosts is typically located on the cell membrane and primarily involved in recognizing various ligands in bacteria [35]. Studies in other fish species like mrigal (*Cirrhinus mrigala*) and rohu (*labeo rohita*) showed that TLR2 expression was induced following exposure to PGN and LTA, as well as Gram-positive or Gram-negative bacterial infection [36,37]. In the orange-spotted grouper (*Epinephelus coioide*), TLR2 expression was also up-regulated in response to LPS, Poly(I:C), and *Vibrio alginolyticus* [38]. The observed up-regulation or down-regulation of NOD1, NOD2, NLRX1, NLRP12, NLRC3, and TLR2 mRNA within 48 h suggests that *Po*PerCs can effectively recognize inactivated *V. anguillarum* and trigger an immune response.

The genes belonging to the major histocompatibility complex family, responsible for the recognition and presentation of foreign antigens, are important components of the vertebrate adaptive immune system [39]. In higher vertebrates, it has been reported that MHC-I molecules bind peptides consisting of 8–11 amino acids, which subsequently interact with TCRs found on CD8^+^ T lymphocytes. Conversely, MHC-II molecules bind longer peptides ranging from 12 to 25 amino acids, and interact with TCRs of CD4^+^ T lymphocytes [40]. There is substantial evidence suggesting that MHC-I and II molecules perform similar functions in both fish and mammals [41]. In the Antarctic bullhead notothen (*Notothenia coriiceps*), it has been observed that inactivated *Escherichia coli* activates genes involved in both MHC-I and MHC-II antigen processing and presentation pathways [42]. Similarly, live attenuated *V. anguillarum* has been shown to up-regulate the expression of genes related to both MHC-I and II in zebrafish (*Danio rerio*) [43]. In our study, we found that inactivated *V. anguillarum* could up-regulate the expression of genes related to the MHC-I pathway *(HSC70*, *MHC-I*, *CANX*, *CD8α*, etc.) and MHC-II (*CTSB*, *MHC-II*, *CD4-1*, etc.) pathway. Interestingly, their expression did not show simultaneous up-regulation. This indicates that inactivated *V. anguillarum* induces a specific immune response in flounder through both MHC-I and II pathways.

Autophagy is a highly conserved self-digestion process and it involves lysosomal degradation of cytoplasmic material [44]. One of its non-canonical functions is its participation in APP for MHC-II activation of CD4^+^ T cells. This activation is through a process known as LC3/GABARAP-associated phagocytosis (LAP), relying on key autophagy-related molecules such as ATG5 and ATG16L1 [45,46]. In mammals, dendritic cells deficient in ATG5 are less efficient at presenting extracellular antigens to CD4^+^ T cells [47]. Moreover, both human and mouse studies have found that ATG16L1 is also essential for the efficient presentation of MHC-II in dendritic cells and macrophages during LAP-mediated antigen processing [48]. In zebrafish, LAP has been identified as the primary host protective autophagy-related pathway responsible for macrophage defense during systemic infection by *Salmonella typhimurium* [49]. In our study, we observed an up-regulation in the expression of autophagy-related genes such as *ATG5*, *ATG16L1*, and *GABARAPL1* after immunization. This expression pattern paralleled that of MHC-II-related genes. Therefore, it is plausible that LAP may promote the MHC-II antigen presentation pathway in flounder as well, and this process may also depend on ATG5 and ATG16L1.

CD4^+^ T cells, also known as Th cells, are integral to coordinating immune responses [50]. They perform multiple functions in fish, such as stimulating B cells to produce antibodies, recruiting granulocytes (neutrophils, eosinophils, and basophils) to the site of inflammation, and modulating immune responses [51,52]. CD4^+^ T cells can be further categorized into several major subpopulations, including Th1, Th2, and Th17, and each possesses a distinct role in immune responses [53]. Th1 cell differentiation is primarily regulated by key cytokines like IFN-γ and IL-12, which act through the control of transcription factors such as master regulator T-bet, STAT1, and STAT4 [54]. Th2 cell differentiation, on the other hand, is driven by cytokines like IL-4/13, which influence several master regulators including GATA-3, STAT5, and STAT6 [55,56]. For Th17 cell differentiation, IL-6 and TGF-β play crucial roles, and along with the two key cytokines lie three master regulators, namely retinoic acid receptor-related orphan receptors (RORα and RORγ) and STAT3 [57]. In our study, we found the expression of key factors associated with Th1 cells (*IFN-γR*, *IL-12R*, *STAT1*, and *STAT4*), Th2 cells (*STAT5B* and *STAT6*), and Th17 cells (*IL-6R*, *TGF-βR*, and *RORα*) were up-regulated within 48 h following immunization. This suggests that inactivated *V. anguillarum* has the potential to induce the differentiation of Th cells in flounder.

## 5. Conclusions

In summary, our study represents the first attempt to conduct a transcriptome analysis of *Po*PerCs in response to immunization with inactivated *V. anguillarum*. We identified a total of 14561 DEGs, suggesting that inactivated *V. anguillarum* triggers a systemic immune response in *Po*PerCs. Based on WGCNA, we identified a hub module closely related to immunization time, characterized their biological functions and highlighted the involved signaling pathways, and identified a series of hub genes. These findings contributed to a deeper understanding of the early-stage immune response to inactivated *V. anguillarum* in flounder. Consequently, our results lay the groundwork for further investigation into the molecular mechanisms underlying the immune response of flounder to inactivated bacterial pathogens.

## Figures and Tables

**Table 1:** Primers used for the qPCR analysis of selected genes.

Name	Sequence (5′-3′)	Accession Number	Amplicon Length (bp)
NOD2-F	TGGTAGGTAATGGTGTAGGGAATG	XM_020079852.1	138
NOD2-R	CCAGGGCTTGAACCAGACTTT		
TLR2-F	CATGGAAACAGAGTAGCTGGGATT	XM_020112938.1	151
TLR2-R	TGTGGAGCAGGTTGAGACGC		
MHC-II-F	CTATCACTATTGTGGGCTGCTTTG	XM_020093263.1	190
MHC-II-R	TGCTCTGCTTTCTTGACACCTTT		
ATG5-F	CCTCCACTGTCCGTCCAACT	XM_020093489.1	256
ATG5-R	CGGTCTATCACTCATCGTCTGG		
GABARAPL1-F	TGTGCTTCCTCATCCGTCAG	XM_020093139.1	126
GABARAPL1-R	CCTCTTCATGGTGCTCCTCATA		
TCR β-F	CCCCACTACATCTCAAGGTTTCC	XM_020105957.1	151
TCR β-R	CAAAGTTTACACTGCTGCCCAC		
CD4-1-F	CCAGTGGTCCCCACCTAAAA	XM_020093150.1	82
CD4-1-R	CACTTCTGGGACGGTGAGATG		
STAT4-F	CCAGCAAAGTCCATCCATACA	XM_020099666.1	151
STAT4-R	TCGAAGCACAGATGCTCGTTT		
RORα-F	CCTTACTGCTCCTTCACCAACG	XM_020079419.1	252
RORα-R	GGCGAACTCCACCACATACTG		
β-actin-F	GAGGGAAATCGTGCGTGACAT	AF135499.1	142
β-actin-R	ATTGCCGATGGTGATGACCTG		

**Table 2:** Summary of transcriptome data from *Po*PerCs samples.

Sample	Raw Data (bp)	Clean Data (bp)	Q20 (%)	Q30 (%)	GC (%)	Unique Mapped (%)	Total Mapped (%)
IV-0-1	7,885,244,100	7,787,759,502	97.16	92.38	47.89	89.53	91.34
IV-0-2	6,677,778,000	6,600,187,229	97.55	93.22	47.92	90.05	91.83
IV-0-3	7,790,959,800	7,684,573,272	97.45	93.05	47.90	89.62	91.43
IV-6-1	8,263,775,700	8,155,916,011	97.35	92.88	47.61	89.33	91.15
IV-6-2	8,468,135,100	8,371,786,696	97.65	93.46	47.64	89.64	91.53
IV-6-3	7,501,817,700	7,405,558,922	97.45	93.10	47.60	89.40	91.19
IV-12-1	9,283,881,900	9,172,219,940	97.47	93.13	47.93	89.96	91.83
IV-12-2	7,436,369,400	7,352,695,274	97.36	92.87	47.75	89.56	91.41
IV-12-3	8,982,513,000	8,889,939,324	97.55	93.19	47.79	89.85	91.7
IV-24-1	10,557,306,600	10,459,182,090	97.38	92.91	48.01	90.01	91.89
IV-24-2	8,221,355,100	8,141,427,164	97.56	93.38	47.61	90.33	92.12
IV-24-3	7,957,429,800	7,864,449,269	97.20	92.46	47.98	90.16	91.97
IV-48-1	6,749,397,000	6,677,417,329	97.46	93.12	47.70	90.04	91.82
IV-48-2	8,024,550,900	7,930,624,583	97.22	92.59	47.40	89.59	91.36
IV-48-3	7,079,832,300	7,002,516,806	97.28	92.62	47.41	89.94	91.66

**Table 3:** Number of genes in each WGCNA module.

Module	Gene Number	Module	Gene Number	Module	Gene Number
Blue	11,616	Magenta	359	Royal blue	238
Brown	2093	Purple	354	Dark red	237
Red	1520	Green–yellow	329	Dark green	230
Green	693	Salmon	300	Dark grey	194
Dark turquoise	506	Cyan	290	Dark orange	180
Black	498	Grey60	260	White	178
Pink	385	Light green	259	Saddle brown	95

## Data Availability

The original contributions presented in the study are included in the article. The raw data of RNA-Seq have been deposited to the NCBI data bank with the accession number PRJNA1012664. Further inquiries can be directed to the corresponding authors.

## Supplementary Materials

The following supporting information can be downloaded at: https://www.mdpi.com/article/10.3390/vaccines11101603/s1, Figure S1: Expression of Th cell differentiation-related genes in intestine, spleen, head kidney, and peripheral blood leukocytes (PBLs) after inactivated *Vibrio anguillarum* immunization.
