# Supplementary material for: Transcriptome Analysis of Peritoneal Cells Reveals the Early Immune Response of Flounder (Paralichthys olivaceus) to Inactivated Vibrio anguillarum Immunization
Source: Vaccines (Basel). 2023 Oct 16;11(10):1603. doi: 10.3390/vaccines11101603 (PMC10611026; doi:10.3390/vaccines11101603)
Supplement: Supplementary file 1 [file vaccines-11-01603-s001.zip › vaccines-2623747-supplementary.pdf]

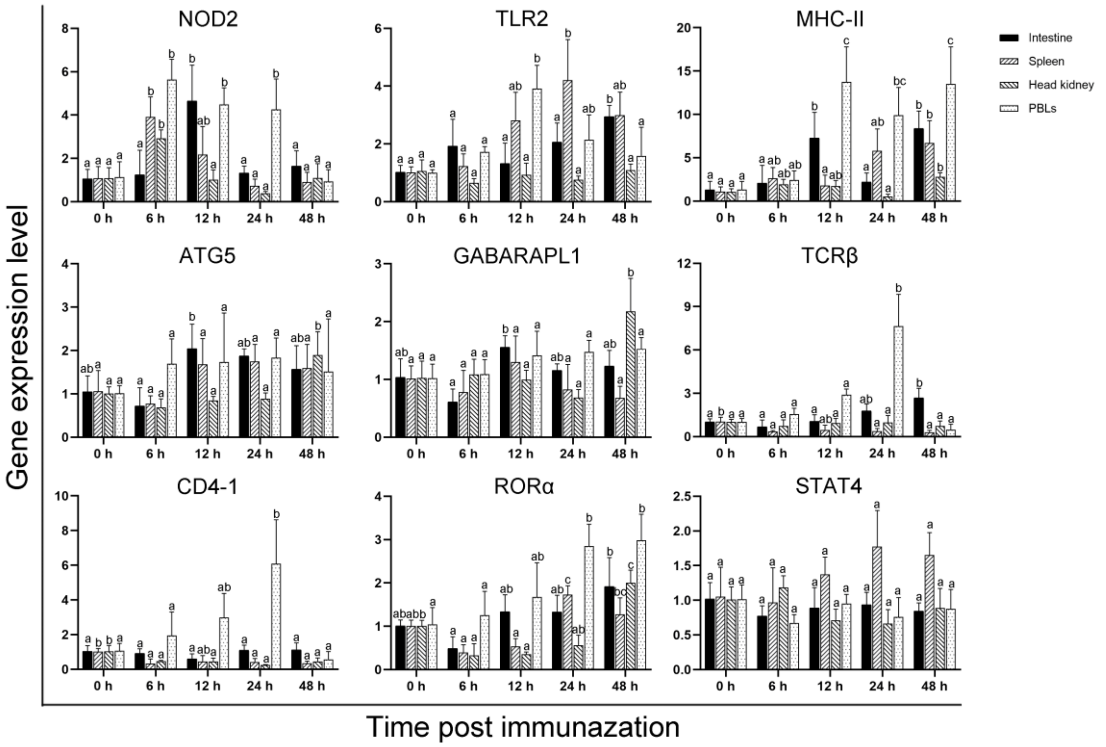

Figure S1. Expression of Th cell differentiation related genes in intestine, spleen, head kidney and peripheral blood leukocytes (PBLs) after inactivated *Vibrio anguillarum* immunization. Results are shown as means ± SD (N = 3). Different letters on the bar represent the statistical significance ($P < 0.05$) of difference between the experimental (6, 12, 24 and 48 hours after immunization) and control groups (0 hour after immunization).
